# Childhood Height Growth Rate Association With the Risk of Islet Autoimmunity and Development of Type 1 Diabetes

**DOI:** 10.1210/clinem/dgac121

**Published:** 2022-03-04

**Authors:** Zhiguo Li, Riitta Veijola, Eileen Koski, Vibha Anand, Frank Martin, Kathleen Waugh, Heikki Hyöty, Christiane Winkler, Michael B Killian, Markus Lundgren, Kenney Ng, Marlena Maziarz, Jorma Toppari

**Affiliations:** 1 Center for Computational Health, IBM T.J. Watson Research Center, Yorktown Heights, 10598 NY, and Cambridge, MA, USA; 2 Department of Pediatrics, PEDEGO Research Unit, University of Oulu, 90014 Oulu, and Oulu University Hospital, Oulu, Finland; 3 JDRF, New York, NY, USA; 4 Barbara Davis Center for Diabetes, University of Colorado, Denver, CO, USA; 5 Department of Virology, Faculty of Medicine and Health Technology, Tampere University, and Fimlab Laboratories, Pirkanmaa Hospital District, Tampere, Finland; 6 Institute of Diabetes Research, Helmholtz Zentrum München, German Research Center for Environmental Health, Munich-Neuherberg, Germany; 7 Forschergruppe Diabetes e.V. at Helmholtz Zentrum, München, German Research Center for Environmental Health, Munich-Neuherberg, Germany; 8 Forschergruppe Diabetes, Technical University Munich, at Klinikum rechts der Isar, Munich, Germany; 9 Pacific Northwest Research Institute, Seattle, WA, USA; 10 Department of Clinical Sciences, Lund University Diabetes Center, Malmö, Sweden; 11 Department of Pediatrics, Kristianstad Hospital, Kristianstad, Sweden; 12 Institute of Biomedicine, Research Centre for Integrative Physiology and Pharmacology, and Centre for Population Health Research, University of Turku, and Department of Pediatrics, Turku University Hospital, Turku, Finland

**Keywords:** type 1 diabetes, autoantibodies, prospective cohort, child growth

## Abstract

**Context:**

Rapid growth has been suggested to promote islet autoimmunity and progression to type 1 diabetes (T1D). Childhood growth has not been analyzed separately from the infant growth period in most previous studies, but it may have distinct features due to differences between the stages of development.

**Objective:**

We aimed to analyze the association of childhood growth with development of islet autoimmunity and progression to T1D diagnosis in children 1 to 8 years of age.

**Methods:**

Longitudinal data of childhood growth and development of islet autoimmunity and T1D were analyzed in a prospective cohort study including 10 145 children from Finland, Germany, Sweden, and the United States, 1-8 years of age with at least 3 height and weight measurements and at least 1 measurement of islet autoantibodies. The primary outcome was the appearance of islet autoimmunity and progression from islet autoimmunity to T1D.

**Results:**

Rapid increase in height (cm/year) was associated with increased risk of seroconversion to glutamic acid decarboxylase autoantibody, insulin autoantibody, or insulinoma-like antigen-2 autoantibody (hazard ratio [HR] = 1.26 [95% CI = 1.05, 1.51] for 1-3 years of age and HR = 1.48 [95% CI = 1.28, 1.73] for >3 years of age). Furthermore, height rate was positively associated with development of T1D (HR = 1.80 [95% CI = 1.15, 2.81]) in the analyses from seroconversion with insulin autoantibody to diabetes.

**Conclusion:**

Rapid height growth rate in childhood is associated with increased risk of islet autoimmunity and progression to T1D. Further work is needed to investigate the biological mechanism that may explain this association.

Rapid linear growth has been suggested as a risk factor for type 1 diabetes (1). According to the accelerator hypothesis (2-4), obesity and rapid weight gain could contribute to development of type 1 diabetes via insulin resistance (5). There are some results supporting this hypothesis (6-8). Rapid linear growth in infancy has been associated with increased risk of islet autoimmunity and type 1 diabetes. The ongoing TEDDY study (8) recently analyzed distinct infant and childhood growth phases in relation to the risk of type 1 diabetes and found that a lower height growth rate in infancy (hazard ratio [HR] = 0.79 [95% CI = 0.70, 0.90] per 1 cm/year) and higher rate in early childhood (after inflection point between infant and childhood growth periods that occurs around 7-10 months of age) (HR = 1.48 [95% CI = 1.22, 1.79] per 1 cm/year) was associated with increased risk of progression from islet autoimmunity to type 1 diabetes. This suggests that growth rates in distinct childhood growth phases might be associated differently with development of islet autoimmunity and progression to clinical type 1 diabetes. The infancy-childhood-puberty (ICP) growth model recognizes different regulatory mechanisms of growth in successive phases (9).

Taking these findings into account, our study focused specifically on the childhood growth phase, for which we have had a unique opportunity to examine the Type 1 Diabetes Intelligence study (T1DI) cohort (10), a large cohort of children prospectively followed for development of islet autoimmunity and diagnosis of type 1 diabetes. Longitudinal data in this cohort represents child growth patterns in the United States and Europe. Infants and toddlers in the T1DI cohort were systematically followed for development of islet autoantibodies starting at very young ages, often as early as 6 months of age. While previous work sheds light on development of islet autoimmunity in early childhood, including infancy, those analyses were restricted to the period up to 4 years of age (7). In this study, we analyze the association of rapid growth during the growth period from early childhood to pre-adolescence, which we define as the growth period from 1 to 8 years of age.

We hypothesized that rapid growth rate in childhood is associated with both development of islet autoimmunity and progression to type 1 diabetes. To test this hypothesis, we analyzed data on 10 145 children from 1 to 8 years of age to evaluate the association between 4 main predictors (height, weight, and rates of change in height and weight) and 2 main outcomes (seroconversion [with 4 types of seroconversion events] or type 1 diabetes) in 3 analysis timeframes (from 1 year of age to confirmed seroconversion to islet autoimmunity; from one year of age to onset of type 1 diabetes; and from seroconversion to onset of type 1 diabetes).

## Methods

### Study Population

The prospective T1DI study cohort consists of 24 662 children who have been followed in 5 natural history studies of type 1 diabetes including 2 in the United States, DAISY (11) and DEW-IT (12), and 3 in Europe, specifically, Germany (BABYDIAB) (13), Sweden (DiPiS) (14), and Finland (DIPP) (15). The focus of these ongoing studies is development of islet autoimmunity and progression to type 1 diabetes in children at high genetic or familial risk. All 5 studies have followed children at least up to 15 years of age or until diagnosis with type 1 diabetes, whichever occurred first.

Supplementary Figure S1 describes the formation of the present study cohort (16). From the full T1DI cohort, we previously analyzed (10) an infant-toddler subcohort restricted to children with first autoantibody measurement by 2.5 years of age (n = 16 709). From this infant-toddler subcohort, we selected children 1.0 to 8.0 years of age who had at least 3 height and weight measurements and at least 1 measurement of each of the 3 islet autoantibodies we considered (those against insulin [IAA], glutamic acid decarboxylase [GADA] and insulinoma-like antigen-2 [IA-2A]). Children diagnosed with type 1 diabetes before 1 year of age were excluded from our analyses. The final dataset, comprising 10 145 children, is hereafter referred to as the “childhood growth cohort.” Of these, 131 (1.3%) developed type 1 diabetes by 8 years of age during follow-up. Baseline data on sex, harmonized human leukocyte antigen (HLA) risk group (10), and study site were available for each subject. Since very few children in DEW-IT were recruited early enough to have the necessary growth and autoantibody data, they were not included in the analysis.

In all studies from which data were included in this analysis, height and weight were measured by trained staff such as clinicians, clinical staff, or research personnel. Measurements were taken using appropriate professional equipment, such as stadiometers or infantometers, and scales with a high degree of precision. In most cases, height was measured with children standing as soon as they were able to do so, which was generally between 1 and 2 years of age.

### Statistical Methods

The HLA typing data was divided into 4 groups based on previously assessed risk of type 1 diabetes (17) and treated as a categorical variable with “HLA group A” being the highest risk group and “HLA group D” the lowest, with the latter treated as the reference in the analyses. Study site was treated as a categorical variable, and sex as binary (1 = male, 0 = female). The missing height (cm) and weight (kg) measurements were first imputed based on multiple imputation methodology using CDC reference which yielded low estimates of error (18). Imputations were then further refined with the best linear unbiased predictors for each subject estimated by linear mixed effects models with height or weight as the outcome, age and quadratic age as the fixed effects, with random intercepts and slopes, and adjusted for sex and study site (Supplementary Materials Supplement 2) (16). We compared the observed and estimated growth trajectories to evaluate the appropriateness of the imputation model (Supplementary Figures S2-S3) (16). Growth rate measurements, specifically, rates of change in height (cm/year) and weight (kg/year), were estimated using the first derivatives of the polynomials in the fitted models. Autoantibody status (1 = positive, 0 = negative) for IAA, GADA, and IA-2A was available from each study site using positivity thresholds appropriate for the assays used in each study. Missing autoantibody status information was imputed for some of the visits based on autoantibody status at previous or later visits in a given timeframe (Supplementary Materials Supplement 3) (16).

The outcomes of interest were events defined in detail below. An event was said to be observed (event = 1) if the outcome of interest was documented for a given subject within the analysis timeframe, otherwise, the observation was censored (event = 0) at the time of the last observed visit within the analysis timeframe or at 8 years of age. The main outcomes were seroconversion and type 1 diabetes, which were used in 3 sets of analyses differing by start time (t_0_) and outcome: (1) 1 year of age (t_0_) to seroconversion; (2) 1 year of age (t_0_) to type 1 diabetes diagnosis; and (3) seroconversion (t_0_) to type 1 diabetes diagnosis. The age at seroconversion with a given autoantibody (or autoantibodies) was defined as the age at the first of 2 consecutive samples positive for that autoantibody (or autoantibodies). In our analysis, we considered 4 seroconversion events: (A) seroconversion to the first of any of IAA, GADA, or IA-2A; (B) seroconversion to IAA; (C) seroconversion to GADA; and (D) seroconversion to multiple autoantibodies, defined as seroconversion with a given autoantibody when at least 1 additional autoantibody was also detected at either or both visits. Note that these 4 seroconversion events are not mutually exclusive. For example, a subject who seroconverted to IAA and GADA in the same visit (ie, became positive for IAA and GADA in the same visit and remained positive for both at least until the next visit), would be present in the 4 analyses for the 4 seroconversion events we considered. We did not analyze seroconversion to IA-2A as the number of subjects with this outcome was too small. Diagnosis of clinical type 1 diabetes was defined according to the American Diabetes Association (ADA) criteria (19) and age at diagnosis was recorded.

To evaluate whether height, weight, rate of change in height, or rate of change in weight were associated with islet autoimmunity or type 1 diabetes during the childhood growth phase, we used Cox regression with time-varying covariates and time-varying coefficients. The 9 models, grouped by the 3 analysis timelines, are defined below and are summarized in Table 1. Further details of subjects in each analysis are given in Supplementary Tables S1-S9 (16).

**Table 1.** Characteristics of 9 models

Model	Start (*t*_*0*_)	Outcome	Islet Autoantibody seroconversion event	Adjustments	Subjects (events) (n)
1A	Age = 1 year	Seroconversion	Any (IAA, GADA, or IA-2A)	Sex, HLA	10 008 (592)
1B			IAA		10 045 (361)
1C			GADA		10 086 (458)
1D			Multiple		10 070 (359)
2	Age = 1 year	Type 1 diabetes	n/a	Sex, HLA, islet autoantibody seroconversion event	10 144 (131)
3A	Seroconversion	Type 1 diabetes	Any (IAA, GADA, or IA-2A)	Sex, HLA	720 (92)
3B			IAA		458 (77)
3C			GADA		508 (70)
3D			Multiple		428 (92)

#### One year of age (t_0_) to seroconversion with seroconversion events A to D

*Model 1A.* Cox model with 1 year of age as the start time of the analysis and seroconversion with the first of any of IAA, GADA, or IA-2A as the outcome, time-dependent covariates for height (cm), weight (kg), rate of change in height (cm/year), rate of change in weight (kg/year), adjusting for sex, HLA, and stratifying by study site.

*Model 1B to 1D.* As Model 1A, but with seroconversion with IAA (Model 1B), GADA (Model 1C), or multiple autoantibodies (Model 1D) as the outcome.

#### One year of age (t_0_) to type 1 diabetes diagnosis

*Model 2.* Cox model with 1 year of age as the start time of the analysis and type 1 diabetes diagnosis as the outcome, time-dependent covariates for height (cm), weight (kg), rate of change in height (cm/year), rate of change in weight (kg/year), adjusting for autoantibody status (binary) of IAA, GADA, and IA-2A modeled as time-dependent covariates, as well as sex, HLA, and stratifying by study site.

#### Age at seroconversion (t_0_) with seroconversion events A to D to type 1 diabetes diagnosis

*Model 3A.* Cox model with age at seroconversion with the first of any of IAA, GADA, or IA-2A as the start time of the analysis and type 1 diabetes as the outcome, time-dependent covariates for height (cm), weight (kg), rate of change in height (cm/year), rate of change in weight (kg/year), adjusting for age at seroconversion, autoantibody status (binary) of each of IAA, GADA, and IA-2A modeled as time-dependent covariates, as well as sex, HLA, and stratifying by study site.

*Model 3B to 3D.* As Model 3A, but with the age at seroconversion with IAA (Model 3B), GADA (Model 3C), or multiple autoantibodies (Model 3D) as the start time of the analysis, and type 1 diabetes diagnosis as the outcome.

After fitting these models, we assessed the validity of the proportional hazards assumption using a proportional hazards test (20). The covariates for which the proportional hazards assumption failed were investigated by plotting Schoenfeld residuals. We noted that the associations between HLA group B and C (vs group D), height, rate of change of height, weight, and the rate of change of weight were associated piecewise-linearly with the outcome in at least one of the models starting from 1 year of age. Specifically, we noted a changepoint in the associations between the outcomes and these covariates around 3 years of age in models 1A to 1D and model 2. For models 3A to 3D, that is, starting at seroconversion, the associations between height, weight, and weight rate and type 1 diabetes were piecewise-linear with a changepoint at 3.3 years from seroconversion. Therefore, we also performed all analyses separately for ages 1-3 years and 3-8 years for models 1A to 1D and 1-3.3 years and 3.3+ years from seroconversion for model 3. The reported *P* values are nominal and not adjusted for multiple comparisons. *P* values below 0.01 were considered statistically significant, and those between 0.01 and 0.05 as marginally statistically significant. All analyses were performed in R v3.6.1 (www.r-project.org), using packages survival (v3.1-8) and survminer (v0.4.6) for the survival analysis.

## Results

The characteristics of the full dataset used in this manuscript are summarized in Table 2. There were 10 145 participants, 61% from Finland and 12% to 13% each from Sweden, Germany, and the United States. The majority of the children had the HLA genotype belonging to risk group B (n = 4941, 48.7%) while 2300 (22.7%) belonged to HLA group D, which served as the reference group in the analysis. The mean (SD) duration of follow-up was 6.9 (1.6) years. During that time the average number of height and weight measurements available per subject was 8.1 (5.1) and 8.2 (5.0), respectively. Additionally, on average, we imputed 1.2 (2.0) measurements for height and 1.1 (2.0) for weight.

**Table 2.** Characteristics of the T1DI Study growth rate analysis dataset (n = 10 145)

Participants by study and country, n (%)	
BABYDIAB, Germany	1324 (13.1)
DAISY, United States	1373 (13.5)
DiPiS, Sweden	1233 (12.2)
DIPP, Finland	6215 (61.3)
Duration of follow-up from 1 to 8 years of age (years), mean (SD)	6.94 (1.61)
Number of growth measures available per subject, mean (SD)	
Height	8.12 (5.05)
Weight	8.24 (5.01)
Number of imputed growth measures per subject, mean (SD)	
Height	1.24 (1.95)
Weight	1.13 (1.96)
HLA group, n (%)	
A	1369 (13.5)
B	4941 (48.7)
C	1535 (15.1)
D	2300 (22.7)
Number of events (n (%))	
Seroconversion with IAA, GADA, or IA-2A	592 (5.8)
Seroconversion with IAA	361 (3.6)
Seroconversion with GADA	458 (4.5)
Seroconversion with multiple persistent autoantibodies	359 (3.5)
Type 1 diabetes	131 (1.3)

### One Year of Age (t_0_ in the analysis) to Seroconversion (Fig. 1)

**Figure 1.**
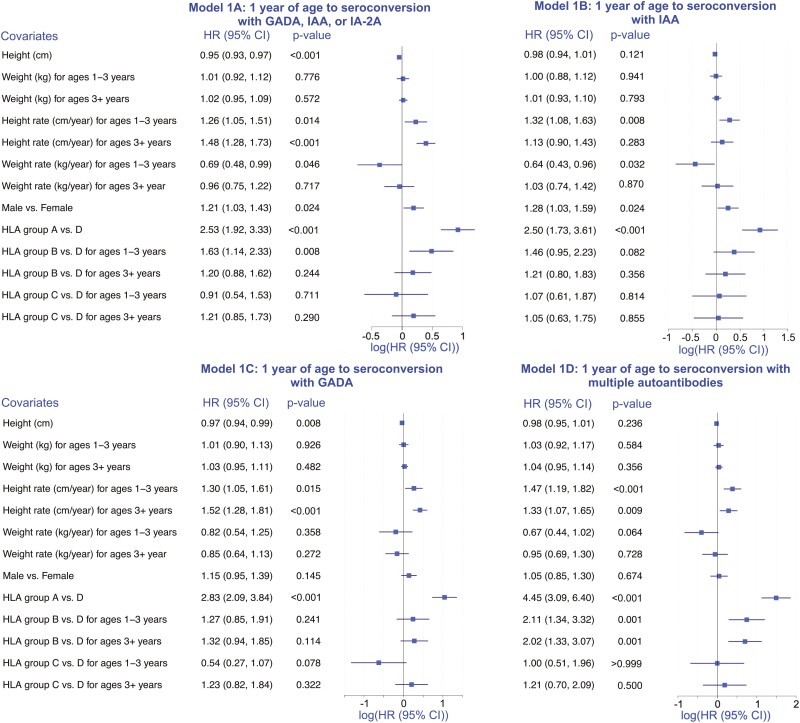
One year of age (t_0_ in the analysis) to seroconversion. Summary of the results of Cox regression analyses to estimate the association between height (cm), weight (kg), and rate of change in height (cm/year) and weight (kg/year), and age at seroconversion with 4 seroconversion types (panels A-D) starting at 1 year of age, adjusting for sex and HLA risk group, and stratifying by study site. The forest plots are a visual representation of log-transformed hazard ratios (HR) and their associated 95% CI reported to the left of the plot in each panel. The panels differ by the outcome. In the top-left panel (Model 1A), the outcome is seroconversion with the first of any of IAA, GADA, or IA-2A; in the top-right panel (Model 1B) the outcome is seroconversion with IAA; in the bottom-left panel (Model 1C), the outcome is seroconversion with GADA; and in the bottom-right panel (Model 1D), the outcome is seroconversion with multiple autoantibodies.

Overall, in this part of the analyses, we noted a statistically significant positive association between each of the outcomes and rate of change in height over time in cm/year (height rate) for children in both the 1-3 years and 3-8 years age groups (Fig. 1A-D). Height was negatively associated with seroconversion with any of IAA, GADA, or IA-2A (Fig. 1A) as well as with GADA (Fig. 1C). The rate of change in weight over time in kg/year (weight rate) in children from 1 to 3 years of age tended to be negatively associated with seroconversion, as evidenced by the HR estimates being consistently < 1, albeit not statistically significantly. In all 4 analyses, we noted statistically significant positive associations between HLA group A (highest risk of type 1 diabetes) vs D (lowest risk) and all outcomes, as well as HLA group B vs D for 2 outcomes with multiple autoantibodies (Fig. 1A and 1C). Additional details on the main results from each of the 4 models (Fig. 1A-1D) are provided below.

The Cox regression analysis from 1 year of age to seroconversion with the first of any of GADA, IAA, or IA-2A as the outcome was based on a sample size of 10 008 and 592 events (Fig. 1A). The estimated HR and the associated 95% CI of the association between height (cm) and the outcome was HR = 0.95 (95% CI = 0.93, 0.97), *P* < 0.001. The rate of change in height (cm/year) was associated with the outcome in children 1-3 years of age (HR = 1.26 [1.05, 1.51], *P* = 0.014) and in children 3-8 years of age (HR = 1.48 [1.28, 1.73], *P* < 0.001) (Fig. 1A).

The analysis from 1 year of age to seroconversion with IAA was based on n = 10 045 and 361 events (Fig. 1B). The rate of change in height (cm/year) was positively associated with seroconversion with IAA in children 1-3 years of age (HR = 1.32 [1.08, 1.63], *P* = 0.008), but not for children 3-8 years of age (HR = 1.13 [0.90, 1.43], *P* = 0.283) (Fig. 1B).

The analysis from 1 year of age to seroconversion with GADA as the outcome was based on n = 10 086 and 458 events (Fig. 1C). Seroconversion with GADA was negatively associated with height (cm) (HR = 0.97 [0.94, 0.99], *P* = 0.008), and positively associated with the rate of change in height (cm/year) in children 1-3 years of age (HR = 1.30 [1.05, 1.61], *P* = 0.015), and in children 3-8 years of age (HR = 1.52 [1.28, 1.81], *P* < 0.001) (Fig. 1C).

Lastly, the analysis from 1 year of age to seroconversion with multiple autoantibodies as the outcome was based on n = 10 070 and 359 events (Fig. 1D). The rate of change in height (cm/year) was positively associated with this outcome in children 1-3 years of age (HR = 1.47 [1.19, 1.82], *P* < 0.001), and in children 3-8 years of age (HR = 1.33 [1.07, 1.65], *P* = 0.009) (Fig. 1D).

### One Year of Age (t_0_) to Type 1 Diabetes (Fig. 2)

**Figure 2.**
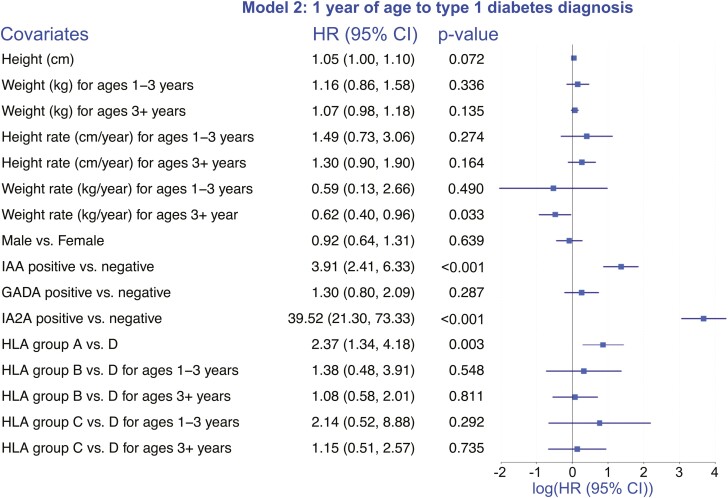
One year of age (t_0_) to type 1 diabetes diagnosis. Summary of the results of Cox regression analysis to estimate the association between height (cm), weight (kg), and rate of change in height (cm/year) and weight (kg/year), and time to type 1 diabetes diagnosis starting at 1 year of age, adjusting for sex, HLA risk group, IAA, GADA, and IA-2A status, and stratifying by study site. The forest plot is a visual representation of log-transformed hazard ratios (HR) and their associated 95% CI reported to the left of the plot.

The Cox regression analysis from 1 year of age to type 1 diabetes as the outcome was based on n = 10 144 subjects and 131 events (Fig. 2). The direction of the association between height (cm) and the outcome was positive, though not statistically significant (HR = 1.05 (1.00, 1.10), *P* = 0.072). The results for the height rate and the weight rate were consistent with those summarized above in “One Year of Age (t0 in the analysis) to Seroconversion” for seroconversion as the outcome. Specifically, the HR estimates for height rate (cm/year) for ages 1-3 and 3-8 were > 1, and those for the weight rate (kg/year) were < 1. However, none of these associations were found to be statistically significant. As before, HLA group A vs D was positively associated with the outcome. Additionally, a positive association between IAA positivity (vs negativity) and development of type 1 diabetes by age 8 years was observed (HR = 3.91 [2.41, 6.22], *P* < 0.001), and similarly between IA-2A positivity (vs negativity) and type 1 diabetes (HR = 39.5 [21.3, 73.3], *P* < 0.001) (Fig. 2).

### Seroconversion With Seroconversion Event A-D (t_0_) to Type 1 Diabetes (Fig. 3)

**Figure 3.**
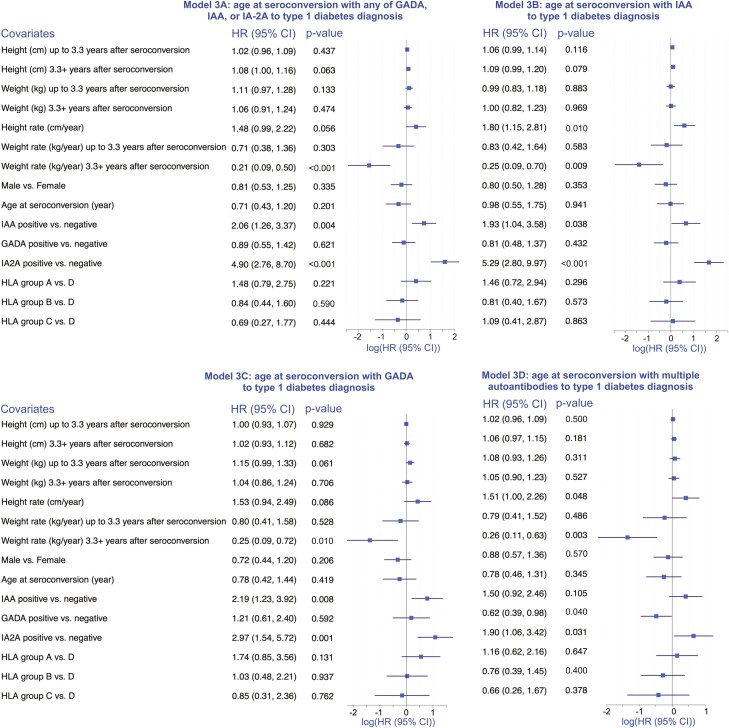
Time of seroconversion (t0 in the analysis) to type 1 diabetes diagnosis. Summary of the results of Cox regression analyses to estimate the association between height (cm), weight (kg), and rate of change in height (cm/year) and weight (kg/year), and time to type 1 diabetes diagnosis, starting from the age at seroconversion with 4 seroconversion types (Models A-D), adjusting for sex, HLA risk group, IAA, GADA, and IA-2A status, age at seroconversion, and stratifying by study site. The forest plots are a visual representation of log-transformed hazard ratios (HR) and their associated 95% CI reported to the left of the plot in each panel. The analyses results summarized in each panel differ by the starting point of the analysis, the outcome in all panels is time to type 1 diabetes. In the top-left panel (Model 3A), the analysis start time is seroconversion with the first of any of IAA, GADA, or IA-2A; in the top-right panel (model 3B), the analysis starts at the time of seroconversion with IAA; the bottom-left panel (Model 3C) summarizes an analysis starting from the age of seroconversion with GADA; and in the bottom-right panel (Model 3D) the analysis start time is age at seroconversion with multiple autoantibodies.

The results in the analyses starting from age at seroconversion up to type 1 diabetes as the outcome were relatively consistent regardless of the type of seroconversion (Fig. 3). The HR estimate for the height rate was > 1 in all 4 analyses but was only marginally statistically significant. The rate of change in weight (in kg/year) was negatively associated with risk of type 1 diabetes, especially for children older than 3.3 years from seroconversion, with HRs ranging from 0.21 to 0.26 in the 4 analyses. The association between a lower rate of weight gain and diagnosis of type 1 diabetes was estimated at HR = 0.21 (0.09, 0.5), *P* < 0.001 after 3.3 years after seroconversion with any of GADA, IAA, IA-2A (Fig. 3A); HR = 0.25(0.09, 0.70), *P* = 0.009 after 3.3 years after seroconversion with IAA (Fig. 3B); HR = 0.25 (0.09, 0.72), *P* = 0.010 after 3.3 years after seroconversion with GADA (Fig. 3C); and HR = 0.26 (0.11, 0.63), *P* = 0.003 after 3.3 years after seroconversion with multiple autoantibodies (Fig. 3D). For all analyses, subjects positive for IA-2A were more likely to be diagnosed with type 1 diabetes compared with those who were negative (Fig. 3A-3D). Similar associations between IAA positivity and development of type 1 diabetes were also seen (Fig. 3A-C). In this analysis, the HLA risk group was not found to be associated with development of type 1 diabetes (Fig. 3).

## Discussion

In this analysis, specifically focusing on the age range 1.0 to 8.0 years, rapid childhood growth was associated with both onset of islet autoimmunity and progression from that to clinical type 1 diabetes. Although the strength of the association varied slightly depending on the type of the first appearing autoantibody, rapid height rate consistently predicted islet autoimmunity, as well as development of diabetes from islet autoimmunity, although less significantly compared with the islet autoimmunity endpoint. The well-known association of HLA class II genotype with the risk of islet autoimmunity and type 1 diabetes was also evident (21) and was taken into account in our analysis of the putative growth effect. Interestingly, height was inversely associated with the risk of autoimmunity, suggesting that subsequent catch-up growth is connected to the risk. This is also consistent with the findings in the TEDDY study, in which lower height rate in infancy and higher height rate in early childhood were associated with more rapid progression from autoimmunity to diagnosis of type 1 diabetes (8). In most previous studies, infant and childhood growth periods have been analyzed together rather than separately, which may explain many inconsistencies in the previous findings.

In contrast to height rate, weight rate was inversely associated with the rate of progression to type 1 diabetes. We hypothesize that this phenomenon is caused by the typical weight loss before the clinical diagnosis. Otherwise, we should expect weight to increase together with height. On the other hand, here the association of rapid linear growth with the disease process cannot be explained by an increase in body weight. It is typical that development of overweight is associated with slightly accelerated height rate, but here the children experienced weight loss due to deteriorating endogenous insulin secretion and gradual development of hyperglycemia and polyuria before the diagnosis. However, there was also an inverse association with weight rate and onset of islet autoimmunity, which cannot be explained by preclinical weight loss and needs further investigation. This was also different from the result of the German Fr1da study which showed that obesity but not overweight was associated with an increased risk of islet autoimmunity (22).

Regulation of growth is described by an infancy-childhood-puberty (ICP) growth model which refers to differential regulatory mechanisms in infancy, childhood, and puberty (9). Infant growth is often considered a continuation of fetal growth that rapidly decelerates during the first year of life, whereafter childhood growth continues until the end of puberty and the pubertal growth spurt overlaps it during the last few years before adult stature is achieved. Nutrition is the main driver of growth in infancy, while growth hormone is the most important factor affecting height development in childhood, and sex hormones induce the pubertal growth spurt (23). Most previous studies on the association of growth with the risk of islet autoimmunity and type 1 diabetes have analyzed infant and childhood growth together. This approach may have obscured features that were specific to these distinct growth periods. The present data strongly point to a link between childhood growth and risk of islet autoimmunity and diabetes. Our results are in agreement with those from the TEDDY study that showed a positive association of accelerated childhood growth with progression to diabetes (8). Interestingly, the same study showed an inverse association between rapid infant growth and progression to diabetes, emphasizing the importance of analyzing growth rates at distinct time periods. It is also in agreement with our finding of negative association of height and development of islet autoimmunity by age 8 years. In the TRIGR study, the risk of islet autoimmunity with multiple autoantibodies was positively associated with the child’s height velocity during the first 2 years of life (24). In the DAISY study, greater height growth velocity of children over 2 years of age was associated with both increased risk of islet autoimmunity and type 1 diabetes (2). Blom et al reported that linear growth was positively associated with diabetes risk in Swedish boys, while the association remained nonsignificant in girls albeit the trend was similar (1). In our analyses, male sex was associated with an earlier onset of islet autoimmunity, but growth rate association was independent of sex.

We do not know whether there is a causal relationship between a rapid growth rate and the risk of islet autoimmunity and further progression to type 1 diabetes. However, rapid growth usually comes with high growth hormone and insulin-like growth factor 1 (IGF-1) levels, which may interact with insulin and glucose metabolism. The roles of growth hormone and IGF-1 are not similar in infancy and childhood, as infant growth is less dependent on growth hormone than childhood growth. We do not have data for IGF-1 in the T1DI cohort in order to evaluate whether there were any associations between IGF-1 levels and the risk of islet autoimmunity or type 1 diabetes. Beyerlein et al measured IGF-1 and insulin-like growth factor binding protein-3 (IGFBP-3) in a subset of children but found no association with the risk of islet autoimmunity (25). In their study, early age at infant peak body mass index (BMI) was inversely associated with the risk of islet autoimmunity, whereas height growth rate from infancy forward was not. In further analyses, rapid increase of BMI from infancy to 3 years of age was positively associated with an increased risk of islet autoimmunity, whereas a pattern of higher height SD score at birth followed by a decrease to average values after 3 years was associated with a reduced rate of islet autoimmunity (26). These latter findings are in agreement with our results, although the study combined data from infancy and childhood growth. In the T1DBIT and DIABIMMUNE studies, lower IGF-1 levels were reported in children with islet autoimmunity and type 1 diabetes than in autoantibody-negative children (27, 28), which may be caused by decreased intraportal insulin levels leading to growth hormone resistance in the liver and thereby to lower IGF-1 production (29). This leads to increased growth hormone secretion due to declined negative feedback by IGF-1, and consequently growth hormone can stimulate paracrine secretion of IGF-1 in the growth plate to stimulate growth. While we speculate that this may be true in this setting, it is known that severe long-standing hyperglycemia and concomitant high levels of growth hormone may be associated with impaired growth, so-called Mauriac syndrome.

Rapid growth presents a high metabolic demand to the beta cells causing endoplasmic reticulum stress and oxidative stress, which have been hypothesized to originate beta cell demise and subsequent autoimmunity (30). This is difficult to measure in vivo and remains therefore hypothetical. However, the clear association between childhood growth rate and the risk of islet autoimmunity and progression to type 1 diabetes gives us an insight into further research needs. New biomarkers of beta cell stress and apoptosis need to be identified and validated.

The strengths of the study are prospective systematic collection of data, its large study population and representation of multiple nationalities, which makes the findings more generalizable. Our study also has some limitations. We were not able to analyze the infant growth phase, which limits comparability to earlier studies covering growth from birth onwards. The T1DI cohort comprises 5 studies, each of which had its own study protocol. Visit schedules varied, as well as inclusion criteria (HLA criteria or family history). Despite these differences, it was possible to combine and harmonize the data to make the analyses described in this manuscript possible.

In conclusion, rapid height growth rate in childhood is associated with increased risk of islet autoimmunity and, among subjects who seroconverted with IAA, with rapid progression to type 1 diabetes. Including rapid childhood growth in risk score models may improve the prediction accuracy of time to islet autoimmunity and type 1 diabetes. Further work is needed to investigate how much improvement in the risk score can be gained, as well as to elucidate the biological mechanism that may explain these associations.

## Data Availability

The data that support the findings of this study are not publicly available because they were used under license for the current study only. Data are, however, available upon reasonable request with permission from the originating sites, whose representatives are William Hagopian (DEW-IT), Markus Lundgren (DiPiS), Marian Rewers (DAISY), Riitta Veijola (DIPP), and Anette Ziegler (BABYDIAB). Zhiguo Li is responsible for the accuracy of the data analysis. The resources used included open-source software; however, the specific code is subject to proprietary constraints but can be made available upon reasonable request to the corresponding author.

## Abbreviations

GADA: glutamic acid decarboxylase autoantibodies
HLA: human leukocyte antigen
IA-2A: islet antigen 2 antibodies
IAA: insulin autoantibodies
IGF-1: insulin-like growth factor 1
T1DI: Type 1 Diabetes Intelligence study

## References

[1] Blom L , Persson L, Dahlquist G. A high linear growth is associated with an increased risk of childhood diabetes mellitus. Diabetologia. 1992;35(6):528-533. 1612225 10.1007/BF00400480

[2] Lamb MM , Yin X, Zerbe GO, et al Height growth velocity, islet autoimmunity and type 1 diabetes development: the diabetes autoimmunity study in the young. Diabetologia. 2009;52(10):2064-2071. 19547949 10.1007/s00125-009-1428-2 PMC2813468

[3] Larsson HE , Hansson G, Carlsson A, et al; DiPiS Study Group. Children developing type 1 diabetes before 6 years of age have increased linear growth independent of HLA genotypes. Diabetologia. 2008;51(9):1623-1630. 18592208 10.1007/s00125-008-1074-0

[4] Hyppönen E , Virtanen SM, Kenward MG, Knip M, Åkerblom HK. Obesity, increased linear growth, and risk of type 1 diabetes in children. Diabetes Care. 2000;23(12):1755-1760. 11128347 10.2337/diacare.23.12.1755

[5] Wilkin TJ . The accelerator hypothesis: weight gain as the missing link between Type I and Type II diabetes. Diabetologia. 2001;44(7):914-922. 11508279 10.1007/s001250100548

[6] Couper JJ , Beresford S, Hirte C, et al Weight gain in early life predicts risk of islet autoimmunity in children with a first-degree relative with type 1 diabetes. Diabetes Care. 2009;32(1):94-99. 18835948 10.2337/dc08-0821 PMC2606838

[7] Elding Larsson H , Vehik K, Haller MJ, et al Growth and risk for islet autoimmunity and progression to type 1 diabetes in early childhood: the environmental determinants of diabetes in the young study. Diabetes. 2016;65(7):1988-1995. 26993064 10.2337/db15-1180 PMC4915577

[8] Liu X , Vehik K, Huang Y, et al; the TEDDY Study Group. Distinct growth phases in early life associated with the risk of type 1 diabetes: the TEDDY study. Diabetes Care. 2020;43(3):556-562. 31896601 10.2337/dc19-1670 PMC7035588

[9] Karlberg J . A biologically-oriented mathematical model (ICP) for human growth. Acta Paediatr. 1989;78(s350):70-94. 10.1111/j.1651-2227.1989.tb11199.x 2801108

[10] Anand V , Li Y, Liu B, et al; T1DI Study Group. Islet autoimmunity and HLA markers of presymptomatic and Clinical Type 1 Diabetes: joint analyses of prospective cohort studies in Finland, Germany, Sweden, and the U.S. Diabetes Care. 2021;44(10):2269-2276. 10.2337/dc20-1836 PMC8929180 34162665

[11] Rewers M , Bugawan TL, Norris JM, et al Newborn screening for HLA markers associated with IDDM: diabetes autoimmunity study in the young (DAISY). Diabetologia. 1996;39(7):807-812. 8817105 10.1007/s001250050514

[12] Wion E , Brantley M, Stevens J, et al Population-wide infant screening for HLA-based type 1 diabetes risk via dried blood spots from the public health infrastructure. Ann N Y Acad Sci. 2003;1005:400-403. 14679100 10.1196/annals.1288.067

[13] Ziegler AG , Hummel M, Schenker M, Bonifacio E. Autoantibody appearance and risk for development of childhood diabetes in offspring of parents with type 1 diabetes: the 2-year analysis of the German BABYDIAB Study. Diabetes. 1999;48(3):460-468. 10078544 10.2337/diabetes.48.3.460

[14] Larsson HE . A Swedish approach to the prevention of type 1 diabetes. Pediatr Diabetes. 2016;17(Suppl 22):73-77. 27411440 10.1111/pedi.12325 PMC5556697

[15] Kupila A , Muona P, Simell T, et al; Juvenile Diabetes Research Foundation Centre for the Prevention of Type I Diabetes in Finland. Feasibility of genetic and immunological prediction of type I diabetes in a population-based birth cohort. Diabetologia. 2001;44(3):290-297. 11317658 10.1007/s001250051616

[16] Li Z , Veijola R, Koski E, et al, T1DI Study Group. Supplemental material for Childhood Height Growth Rate Association with the Risk of Islet Autoimmunity and Development of Type 1 Diabetes. Posted February 2, 2022. 10.5281/zenodo.5948737 PMC9113806 35244713

[17] Erlich H , Valdes AM, Noble J, et al, for the Type 1 Diabetes Genetics Consortium. HLA DR-DQ Haplotypes and genotypes and type 1 diabetes risk: analysis of the type 1 diabetes genetics consortium families. Diabetes. 2008;57(4):1084-1092. 18252895 10.2337/db07-1331 PMC4103420

[18] Li Z , Toppari J, Lundgren M, et al ; T1DI study group. Imputing Longitudinal Growth Data in International Pediatric Studies: Does CDC Reference Suffice? AMIA Annu Symp Proc. 2022;2021:754-762. 35308906 PMC8861671

[19] American Diabetes Association. Diagnosis and classification of diabetes mellitus. Diabetes Care. 2014;37(Supplement 1):S81-S90. 24357215 10.2337/dc14-S081

[20] Grambsch PM , Therneau TM. Proportional hazards tests and diagnostics based on weighted residuals. Biometrika. 1994;81(3):515-526.

[21] Ilonen J , Kiviniemi M, Lempainen J, et al; Finnish Pediatric Diabetes Register. Genetic susceptibility to type 1 diabetes in childhood - estimation of HLA class II associated disease risk and class II effect in various phases of islet autoimmunity. Pediatr Diabetes. 2016;17(Suppl 22):8-16. 27411431 10.1111/pedi.12327

[22] Ziegler A-G , Kick K, Bonifacio E, et al; Fr1da Study Group. Yield of a public health screening of children for islet autoantibodies in Bavaria, Germany. JAMA. 2020;323(4):339-351. 31990315 10.1001/jama.2019.21565 PMC6990943

[23] Tse WY , Hindmarsh PC, Brook CG. The infancy-childhood-puberty model of growth: clinical aspects. Acta Paediatr Scand Suppl. 1989;356:38-43; discussion 44. 2683573 10.1111/j.1651-2227.1989.tb11238.x

[24] Pacaud D , Nucci AM, Cuthbertson D, et al; TRIGR investigators. Association between family history, early growth and the risk of beta cell autoimmunity in children at risk for type 1 diabetes. Diabetologia. 2021;64(1):119-128. 33026463 10.1007/s00125-020-05287-1 PMC7716821

[25] Beyerlein A , Thiering E, Pflueger M, et al Early infant growth is associated with the risk of islet autoimmunity in genetically susceptible children. Pediatr Diabetes. 2014;15(7):534-542. 24785566 10.1111/pedi.12118

[26] Yassouridis C , Leisch F, Winkler C, Ziegler A-G, Beyerlein A. Associations of growth patterns and islet autoimmunity in children with increased risk for type 1 diabetes: a functional analysis approach. Pediatr Diabetes. 2017;18(2):103-110. 26890567 10.1111/pedi.12368

[27] Shapiro MR , Wasserfall CH, McGrail SM, et al Insulin-like growth factor dysregulation both preceding and following type 1 diabetes diagnosis. Diabetes. 2020;69(3):413-423. 31826866 10.2337/db19-0942 PMC7034187

[28] Peet A , Hämäläinen A-M, Kool P, Ilonen J, Knip M, Tillmann V; DIABIMMUNE Study Group. Early postnatal growth in children with HLA-conferred susceptibility to type 1 diabetes. Diabetes Metab Res Rev. 2014;30(1):60-68. 24038878 10.1002/dmrr.2449

[29] Nambam B , Schatz D. Growth hormone and insulin-like growth factor-I axis in type 1 diabetes. Growth Horm IGF Res. 2018;38:49-52. 29249623 10.1016/j.ghir.2017.12.005

[30] Eizirik DL , Colli ML. Revisiting the role of inflammation in the loss of pancreatic β-cells in T1DM. Nat Rev Endocrinol. 2020;16(11):611-612. 32860020 10.1038/s41574-020-00409-6

